# Retention of CD19 intron 2 contributes to CART-19 resistance in leukemias with subclonal frameshift mutations in CD19

**DOI:** 10.1038/s41375-019-0580-z

**Published:** 2019-10-07

**Authors:** Mukta Asnani, Katharina E. Hayer, Ammar S. Naqvi, Sisi Zheng, Scarlett Y. Yang, Derek Oldridge, Fadia Ibrahim, Manolis Maragkakis, Matthew R. Gazzara, Kathryn L. Black, Asen Bagashev, Deanne Taylor, Zissimos Mourelatos, Stephan A. Grupp, David Barrett, John M. Maris, Elena Sotillo, Yoseph Barash, Andrei Thomas-Tikhonenko

**Affiliations:** 1 Division of Cancer Pathobiology, Children’s Hospital of Philadelphia, Philadelphia, PA 19104 USA; 2 DBHi Bioinformatics Group, Children’s Hospital of Philadelphia, Philadelphia, PA 19104 USA; 3 Division of Oncology, Children’s Hospital of Philadelphia, Philadelphia, PA 19104 USA; 4 Department of Pathology & Laboratory Medicine, Perelman School of Medicine at the University of Pennsylvania, Philadelphia, PA 19104 USA; 5 Department of Genetics, Perelman School of Medicine at the University of Pennsylvania, Philadelphia, PA 19104 USA; 6 Department of Pediatrics, Perelman School of Medicine at the University of Pennsylvania, Philadelphia, PA 19104 USA; 7 Present Address: Lonza Biologics, Portsmouth, NH USA; 8 Present Address: Laboratory of Genetics and Genomics, National Institute on Aging, NIH, Baltimore, MD USA; 9 Present Address: Stanford Cancer Institute, Stanford University School of Medicine, Stanford, CA USA

**Keywords:** Leukaemia, Cancer genetics

Every successful cancer therapy story has Exhibit B, comprised of patients who either did not respond to the initial treatment or acquired resistance after a seemingly curative intervention. The CD19-directed chimeric antigen receptor-armed T-cell therapy (commonly known as CART-19) is the case in point. Although it has revolutionized treatment for B-cell acute lymphoblastic leukemia (B-ALL) in children and adults and gained swift FDA approval, ~30% of patients relapse after complete responses, most often via the loss of the cognate CD19 epitope [1]. Under selective pressure of CART-19 some of MLL-rearranged B-ALL have the propensity to trans-differentiate into myeloid lineages with concomitant loss of CD19 expression [reviewed in [2]]. Transport of CD19 to the plasma membrane is another potentially vulnerable process [3] requiring the dedicated CD81 chaperone; and dysregulation of this gene was reported to contribute to resistance to another CD19-targeted immunotherapeutic blinatumumab [4]. Still, focal alterations of the CD19 gene and its transcript appear to play central role in resistance [5].

In December 2015 our group published in Cancer Discovery the first report addressing the mechanism of resistance to CART-19, based on the analysis of the first 4 patients enrolled in the clinical trial at the Children’s Hospital of Philadelphia [6]. We performed whole exome and RNA sequencing and immunoblotting on relapsed leukemias lacking the CART-19 epitope on the cell surface. We observed that “genetic alterations… accounted for CD19 protein loss in some… but not in other… samples”. For example, in sample CHOP101R we discovered frameshift mutations in exons 2 and 4. However, both were subclonal and collectively accounted for no more than 50% of CD19 alleles, implying the existence of additional mechanisms of gene inactivation. Indeed, we discovered prominent splicing alterations involving increased skipping of exon 2 and exons 5–6 in relapsed leukemias; these pre-existing isoforms were later reported by others as well [7]. Of note, exon 2 is absolutely essential for the integrity of the CART-19 epitope [3] and exons 5–6 encode the CD19 transmembrane domain needed for presentation on the cell surface. Thus, our paper was entitled “Convergence of acquired mutations and alternative splicing of CD19 enables resistance to CART-19 immunotherapy”, stressing the importance of both DNA- and RNA-based mechanisms. As our study included only a small number of patients, we eagerly anticipated the results of additional analyses from the phase II CTL019 clinical trials.

In October 2018, Orlando et al. reported in Nature Medicine on 12 patients with CD19-negative relapses [8]. Their study also incorporated whole-exome DNA-seq and RNA-seq from matching screening and relapse samples. The authors discovered de novo genetic alterations in exons 2–5 in 12 out of 12 samples and loss-of-heterozygosity in 8 out of 9 evaluable patients. They concluded that “homozygous or biallelic frameshift mutations in CD19 are the main source of CD19 loss and acquired resistance to CTL019”.

However, while reviewing data in Orlando et al., we observed that some of the reported mutations were in their own estimate subclonal, even after adjusting for tumor content by means of flow cytometry (patients #2, #5, and #9 in their Supplementary Table 4; gating strategy in their Supplementary Fig. 4). For example, the allelic frequency (AF) of the Q90fs indel in exon 2 of the relapse sample 5R was estimated to be mere 36% and taken at face value could not account for the complete loss of surface CD19 expression, as evidenced by flow cytometry performed on bone marrow cells in Supplementary Fig. 2. In fact, it was reminiscent of the exon 2 frameshift mutation G67fs with AF = 28% in patient CHOP101R from Sotillo et al. Yet, per Orlando et al. there was no evidence for exon 2 skipping in the 5R sample.

**Fig. 1:**
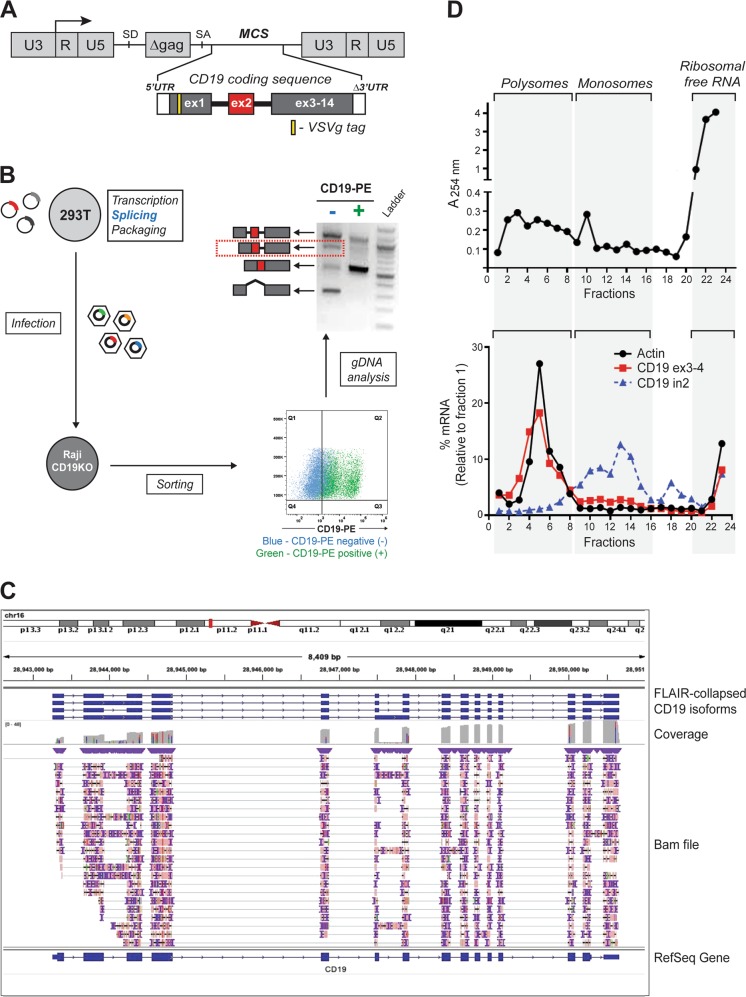
Robust retention of intron 2 limits CD19 protein expression. **a** Schematic representation of the retroviral construct containing N-terminal VSVg-tagged CD19 coding sequence including the two introns flanking exon 2. U3, R, and U5 form the retroviral long terminal repeats (LTR); SD and SA denote 5′ (“donor”) and 3’ (“acceptor”) splice sites, respectively; MCS denotes the multiple cloning site. **b** Splicing of the retrovirally encoded CD19 transcript. The CD19 retroviral cassette was transfected into 293T cells, transcribed, spliced, and packaged into viral particles, which were used to infect a CD19-null derivative of the Raji B-cell line. The transduced Raji cells were sorted into CD19-positive and negative populations after gating based on the parental cell line. The splicing pattern of CD19 exon 2 was analyzed by genomic DNA PCR using the VSVg-specific forward primer to prevent amplification of the endogenous CD19 locus. The identity of all PCR products was confirmed by Sanger sequencing. **c** Long-read RNA-Seq analysis of the endogenous CD19 transcript using the Oxford Nanopore Technology (ONT). The library was prepared from 1 µg of poly(A)-enriched mRNA from REH B-ALL cells using Direct RNA sequencing kit (SQK-RNA002). It was loaded onto a R9.4 flow cell and sequenced on the MinION device FLO-MIN106D for 48 h. The fast5 files were processed using the Guppy algorithm (v3.2.2). Alignment to the human genome (hg19) was achieved using minimap2 (v2.17-r941). The FLAIR package (v1.4) was used to create a collapsed view of the different CD19 isoforms. The structure of the CD19 transcript was visualized using Integrative Genomic Viewer (IGV). The corresponding fastq file has been deposited in GEO under accession number GSE136068. **d** Profiling of ribosomes on the endogenous CD19 transcript. The whole cell lysate was prepared by incubating 2 × 10^7^ REH cells in lysis buffer supplemented with 100 µg/ml cycloheximide. It was layered onto a 10-50% sucrose density gradient, subjected to centrifugation at 35,000 rpm for 3 h, and fractionated into twenty four 0.5-ml fractions. The top panel shows the ribosomal content measured at 254 nm. The bottom panel shows the relative distribution of specific transcripts across the gradient calculated using the formula 2^Ct (fraction 1-X)^ X 100/sum

To better understand the nature of alternative splicing of the CD19 transcript, we generated a retroviral cassette containing the entire CD19 coding sequence in which exon 2 was flanked by introns 1 and 2 (Fig. 1a). This cassette was transfected into 293T cells, where transcription, splicing, and packaging into viral particles took place. To analyze the predominant CD19 splicing isoforms without relying on artifact-prone in vitro reverse transcription reactions, the viral particles were used to infect the B-cell line Raji where the endogenous CD19 had been knocked out using the CRISPR/Cas9 technology and the structure of integrated proviruses was reflective of splicing in 293T cells (Fig. 1b, left). Then the transduced Raji cells were sorted into CD19-positive- and -negative populations (Fig. 1b, bottom right), which called to mind CART-19-sensitive and resistant samples, respectively. We analyzed processing of exon 2 of the CD19 transgene by genomic PCR and Sanger sequencing. We observed that in CD19-positive cells exon 2 was mostly processed correctly. In contrast, CD19-negative populations, in addition to exon 2 skipping, exhibited robust retention of intron 2 (alone or along with intron 1; Fig. 1b, top right), placing a nonsense codon 40 amino acids downstream of the exon 2-intron 2 junction.

To prove that intron retention (IR) occurs in the context of full-length CD19 transcripts and is functionally equivalent to a nonsense mutation, we first performed long-read direct RNA sequencing using the Oxford Nanopore Technology (ONT) [9] and the FLAIR analysis package [10] on several B-cell lines. As shown in Fig. 1c for the REH B-ALL cells, intron 2- retaining mRNAs can account for over a third of full-length reads representing CD19 transcripts. We further observed that unlike properly spliced mRNAs, intron 2 transcripts are found predominantly in the monosome, not polysome fraction (Fig.1d), consistent with the presence of a premature termination codon in intron 2.

**Fig. 2:**
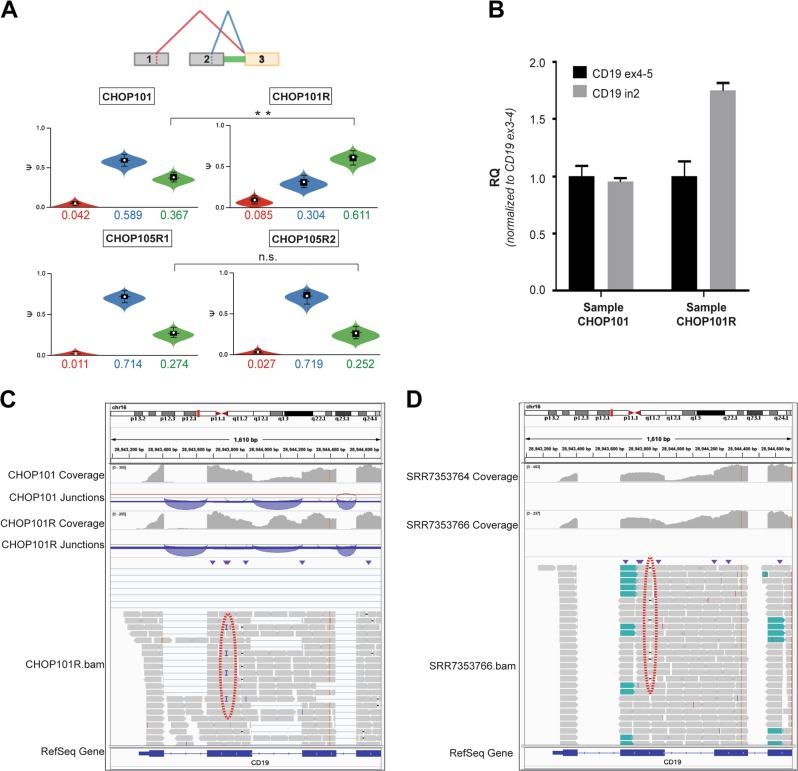
Contribution of CD19 intron 2 retention to resistance to CART-19. **a** MAJIQ output for screening samples CHOP101 and CHOP105R1 compared with relapse samples CHOP101R and CHOP105R2 (top and bottom, respectively). The diagram at the top represents key splicing events. The color-matched violin plots represent the abundance of individual splice isoforms. Double asterisk denote the 0.99 probability of at least 5% difference in percent-spliced-in values (∆psi). **b** RT-qPCR analysis of CD19 intron 2 retention in CHOP101 and CHOP101R samples using oligonucleotides that span constitutive exon/exon junctions (ex4–5 and ex3–4) and a cassette exon/intron junction (ex2-in2). The RQ values were normalized for CD19 expression levels measured using ex3–4 primers, as described previously by Sotillo et al. **c** IGV visualization of CD19 transcripts in CHOP101 and CHOP101R samples using as sources untrimmed bam files. Coverage tracks show read coverage for a given gene segment. Junction tracks summarize reads spanning junctions denoted by arches. The red dotted oval denotes the subclonal frameshift insertion. **d** IGV visualization of CD19 transcripts in 5S and 5R samples using as sources trimmed bam files deposited in the Short Reads Archive as SRR7353764 and SRR7353766. The red dotted oval denotes the subclonal frameshift mutation. Other designations are as in **c**

The apparent importance of this IR event for CD19 expression prompted us to re-evaluate the extent of alternative splicing in the CHOP101R leukemia and its matching screening sample CHOP101. We ran the updated (2.0) version of the previously used MAJIQ algorithm, which in addition to exon skipping and inclusion supports detection of IR events [11]. We again observed an increase in exon 2 skipping described in our 2015 publication (Fig. 2a, top, the “red” event), but we also identified a much more prevalent out-of-frame intron 2 retention event, which would cause premature termination and either nonsense-mediated decay of the transcript or a truncated CD19 protein. Of note, the IR frequency increased appreciably in CHOP101R vs CHOP101 and in fact accounted for ~61% of all reads connected to exon 3 (Fig. 2a, top, the “green” event). Importantly, per MAJIQ, there was no increase in intron 2 retention in the CHOP105R1/R2 diagnosis/relapse pair, where resistance is driven by the indel with a ~100% AF [6] (Fig. 2a, bottom). To increase confidence in our conclusions, we additionally used the independent splicing algorithm rMATS [12] utilized by Orlando et al. (v 4.0.2). rMATS also revealed an increase in intron 2 retention only in the CHOP101/101R comparison (*p* = 0.025), although this difference was not significant after correction for multiple testing due to the low number of samples (data not shown). Finally, we were able to validate increase in intron 2 retention using qPCR with exon/intron-specific primers (Fig. 2b), and the exon2-intron2 boundary was confirmed by next generation sequencing (the Amplicon-EZ protocol).

Armed with this information, we re-analyzed samples 5 and 5R from Orlando et al. deposited to the NCBI Sequence Read Archive (SRA) under the identifier SRP141691. That submission contained aligned DNA-seq and RNA-seq reads corresponding to CD19, CD10, CD22, CD34, CD38, and CD45 genes. We were unable to re-run MAJIQ on these samples, since the deposited BAM files had undergone the Split’N’Trim step recommended by the Genome Analysis Toolkit (GATK) for calling variants in RNA-seq. This module splits junction-spanning reads into its exon segments, which is necessary for optimal variant calling but at the same time precludes the quantitative analysis of splicing events. Thus, we resorted to direct examination of sequencing reads in the IGV browser [13], with samples CHOP101/101R from Sotillo et al. used for comparison. Upon such examination, the increase in IR was apparent in the CHOP101R (Fig. 2c) and 5R samples (Fig. 2d)—and most other samples depicted by Orlando et al. in Supplementary Fig. 1. Admittedly, this includes a control CD19-positive relapse (patient #1). However, even CD19-positive relapses had been under strong selective pressure of CART therapy responsible for initial responses. Thus, increased IR in both groups attests to the importance of this mechanism.

In summary, we are gratified that studies on resistance to CART-19 continue to emerge from clinical trials. In our opinion, the important and timely data by Orlando et al. are broadly consistent with and expand the conclusions from the 2015 paper by Sotillo et al. and have the potential to improve our understanding of the varied mechanisms of resistance to immunotherapy. As sequencing of both DNA and RNA from tumor samples becomes more accessible and affordable, correlative and mechanistic studies on therapeutic resistance will undoubtedly grow explosively, including studies on mRNA processing [14]. However, challenges beyond logistics and costs exist and could be divided broadly into three categories.

One of the biggest sources of data variability is inconsistent tissue procurement and sample collection. Inevitably, clinical scenarios arise that preclude performing analyses on tissues of the same origin or at the same timepoint. For example, for the above-referenced patient #5 in Orlando et al., flow cytometry for CD19 was performed on bone marrow aspirates, but sequencing was carried out on separately collected peripheral blood mononuclear cells, thus confounding the analyses of subclonal mutations. Furthermore, samples from large multi-centric clinical trials are accrued across multiple institutions, which might employ different tumor cell enrichment procedures (Ficoll gradients, flow sorting, etc) and quality control steps (e.g., enumerating blast percentages.) The issue of sampling inconsistency could become especially problematic in longitudinal studies, where the prevalence of clonal mutations could change considerably in the face of therapeutic pressures. This has been particularly well-documented in chronic lymphocytic leukemia [15], but could play a role in B-ALL as well. Thus, adherence to standardized protocols for sample collection and tumor cell enrichment will be increasingly critical as more and more samples are processed across multiple institutions but ultimately need to be combined for large dataset analyses.

Secondly, prolonged storage of leukemia samples at room temperature before RNA isolation introduces biases in gene expression and alters observed splicing patterns independently of RNA integrity, with the most profound changes reported in transcripts with IR and/or subject to nonsense-mediated decay [16]. Even if samples are obtained and processed consistently, one is likely to encounter significant batch effects; and the computational tools to account for them are just beginning to emerge [17].

The last major source of variability is the splicing analysis itself. Currently, there is a wealth of available splicing software, each with its own statistical underpinning and none universally accepted as the *lingua franca* of alternative splicing. While here our group used the latest iteration of MAJIQ, Orlando and co-authors had chosen rMATS for their analyses [12]. In our own experience (and in line with previous publications [18]), there is a considerable overlap, but also a significant variability in their outputs, even when the two were run side-by-side on the same dataset [19, 20]. These unavoidable variances underscore the importance of orthogonal experimental validations (RT-PCR, long-read RNA-seq, etc) and also of open data access. Such access would enable resolution of any discrepancies, increase reproducibility, and serve the interests of the research community and above all - the cancer patients.
